# European Portuguese-Learning Infants Look Longer at Iambic Stress: New Data on Language Specificity in Early Stress Perception

**DOI:** 10.3389/fpsyg.2020.01890

**Published:** 2020-08-28

**Authors:** Sónia Frota, Joseph Butler, Ertugrul Uysal, Cátia Severino, Marina Vigário

**Affiliations:** ^1^Center of Linguistics, School of Arts and Humanities, University of Lisbon, Lisbon, Portugal; ^2^Research and Enterprise Development, University of Bristol, Bristol, United Kingdom; ^3^Faculté des Sciences Économiques, Université de Neuchâtel, Neuchâtel, Switzerland

**Keywords:** infant stress perception, iambic stress, eye-tracking, anticipatory looking, phonology and phonetics of stress, rhythm, frequency

## Abstract

The ability to perceive lexical stress patterns has been shown to develop in language-specific ways. However, previous studies have examined this ability in languages that are either clearly stress-based (favoring the development of a preference for trochaic stress, like English and German) or syllable-based (favoring the development of no stress preferences, like French, Spanish, and Catalan) and/or where the frequency distributions of stress patterns provide clear data for a predominant pattern (like English and Hebrew). European Portuguese (EP) is a different type of language, which presents conflicting sets of cues related to rhythm, frequency, and stress correlates that challenge existing accounts of early stress perception. Using an anticipatory eye movement (AEM) paradigm implemented with eye-tracking, EP-learning infants at 5–6 months demonstrated sensitivity to the trochaic/iambic stress contrast, with evidence of asymmetrical perception or preference for iambic stress. These results are not predicted by the rhythmic account of developing stress perception, and suggest that the language-particular phonological patterns impacting the frequency of trochaic and iambic stress, beyond lexical words with two or more syllables, together with the prosodic correlates of stress, drive the early acquisition of lexical stress. Our findings provide the first evidence of sensitivity to stress patterns in the presence of segmental variability by 5–6 months, and highlight the importance of testing developing stress perception in languages with diverse combinations of rhythmic, phonological, and phonetic properties.

## Introduction

Word stress is a prosodic dimension that varies across languages in two important domains. The first domain relates to the properties of stress in relation to the sound patterns of the language (i.e., the phonological grammar), with languages presenting either variable stress (e.g., Catalan, English, Spanish, and Russian), fixed stress (e.g., Hungarian, Finnish, Polish, and Turkish), or no lexical stress (e.g., French and Korean). In the former languages, the position of stress in a word is not predictable, whereas in the second group of languages stress occurs mostly in a particular position. Only non-predictable stress may be contrastive, i.e., differences in stress pattern can change the meaning of a word as in *insight* /ˈɪnsaɪt/ vs. *incite* /ɪnˈsaɪt/ in English (Peperkamp et al., 2010; Rahmani et al., 2015). The other domain of cross-linguistic variation is the correlates of stress (Lehiste, 1970; Chrabaszcz et al., 2014). The main cues to stress are pitch, duration, intensity, and vowel quality, and the weighting of cues for stress prominence varies between languages. Generally, higher pitch, longer duration, greater intensity, and full (or unreduced) vowels tend to be found in stressed syllables in comparison with unstressed syllables. Importantly, stress has been shown to play a key role in language processing and language acquisition (Cutler, 2012). European Portuguese (EP) is a language with variable stress, which is mainly cued by duration and vowel quality. EP adult speakers, however, have been reported to be unable to perceive stress contrasts in the absence of vowel quality cues, a behavior characteristic of speakers of languages with fixed stress or no lexical stress (Correia et al., 2015). This article presents the first study of the development of stress perception in EP, by examining the perception of trochaic (stress-initial) and iambic (stress-final) stress patterns by EP-learning 5–6-month-old infants.

### Background

There is converging evidence suggesting that infants are equipped with an input processing mechanism initially tuned to prosodic information (e.g., Morgan, 1986; Morgan and Demuth, 1996; Jusczyk, 1997; Höhle, 2009), and prosodic information at the word-level, such as word stress, has been suggested to facilitate language acquisition. Infants may utilize stress to begin developing the ability to segment the speech signal into words (Jusczyk et al., 1999; Nazzi et al., 2006; Shukla et al., 2011; Polka and Sundara, 2012), and to segment the speech signal into phrases (Christophe et al., 2003; Gout et al., 2004; Bion et al., 2011). Stress may also be important for word categorization (Shi et al., 2006) and for word-level and phrase-level meanings (Curtin, 2009, 2010; Frota et al., 2012; Butler et al., 2016), and can even be an early marker of later language abilities (typical or impaired – Weber et al., 2005; Friedrich et al., 2009). These previous studies have demonstrated the importance of the development of stress perception in infancy for language acquisition.

Differences across languages have been reported, whether for stress discrimination abilities or in the acquisition of native stress patterns (Bhatara et al., 2018, for a review). Discrimination abilities have been studied in the absence of segmental variability or in contexts with limited segmental variability, i.e., when infants are presented with a single item (for example, /gaba/ realized either with a trochaic /ˈgaba/ or iambic /gaˈba/ stress pattern), or with tokens only with variation in consonants. Discrimination of stress contrasts is evident in these contexts. This has been shown for Italian newborns (Sansavini et al., 1997), for English-learning infants at 2 months (Jusczyk and Thompson, 1978), for German-learning and French-learning infants at 4–6 months (Weber et al., 2004; Friederici et al., 2007; Höhle et al., 2009; Skoruppa et al., 2013), Spanish-learning infants at 6 months (Skoruppa et al., 2013), and French-learning infants at 9/10 months (Skoruppa et al., 2009; Bijeljac-Babic et al., 2012). Thus, similar early stress discrimination abilities in the absence of full segmental variability are shown by infants learning variable stress languages (English, German, Spanish, and Italian) and languages with no lexical stress (French). However, bilinguals learning French and a variable stress language display better stress discrimination abilities than French-learning monolinguals (Bijeljac-Babic et al., 2012). A different picture emerges when stress discrimination is tested in contexts with segmental variability, which are closer to the phonetic variability found in speech, namely lists of segmentally different words (for example, /ˈdatu/, /ˈnuki/, etc., with trochaic stress, and /daˈtu/, /nuˈki/, with iambic stress). In these contexts, younger infants have difficulties in discriminating stress patterns, as shown by Italian newborns (Sansavini, 1997) and Spanish and French-learning 6-month-olds (Skoruppa et al., 2013). Similarly, English-learning 6-month-olds show no preference for the predominant stress pattern (trochaic stress) of their native language (Jusczyk et al., 1993). By contrast, 8–12-month-old infants are able to discriminate stress patterns if they are learners of a variable stress language, as shown by Spanish-learning and English-learning infants (Skoruppa et al., 2009, 2011), and English learners already show a preference for the predominant stress pattern of English (Jusczyk et al., 1993). However, French-learning 9/10-month-olds, who are learning a language without lexical stress, continue to show no discrimination abilities (Skoruppa et al., 2009; Abboub et al., 2015). This contrasts with bilinguals learning French and a variable stress language, who, unlike French-learning monolinguals, are able to successfully discriminate stress patterns in contexts with segmental variability (Abboub et al., 2015). Crucially, in the presence of segmental variability, discrimination is only evident in learners of variable stress languages. This indicates that only learners of variable stress languages are able to abstract and generalize the contrastive stress patterns across the phonetic variability shown by lists of segmentally different words. The processing of stress is particularly relevant in these languages, given that variability in stress position is part of the phonological grammar allowing stress to be used contrastively to signal word meanings. Thus, infants’ sensitivity to lexical stress contrasts probably reflects the acquisition of the phonological grammar of the native language.

Infants’ sensitivity to stress may be manifested by patterns of asymmetrical perception or stress preferences that favor one of the stress patterns over the other. Asymmetrical perception or stress preference indicates that infants process trochaic and iambic stress differently, by being more sensitive or more attentive to one of the patterns. This advantage of one of the stress patterns has generally been linked to the language-specific development of the acquisition of stress. Asymmetrical stress perception, to the best of our knowledge, has only been reported in event-related potentials (ERPs) studies, in the absence of segmental variability, and may emerge as early as 4–5 months (Weber et al., 2004; Friederici et al., 2007). It is thus not fully understood how it may relate to behavioral findings on stress preferences. However, it is possible that asymmetry in perception is an indication of preference. For example, German-learning infants have been shown to develop an asymmetrical perception favoring trochaic stress between 4 and 5 months (Weber et al., 2004) and a preference for trochaic stress between 4 and 6 months (Höhle et al., 2009). In a behavioral study on the perception of the intonation contrast between statements and yes-no questions, it was found that English-learning infants demonstrated an asymmetrical perception favoring questions, a find that was related to a general preference for high/rising pitch (Soderstrom et al., 2011). The emergence of preferences for stress patterns has been studied both in contexts with no segmental variability and with segmental variability. While a preference for the trochaic pattern has been found to develop between 4 and 6 months of age for German-learning infants, French-learning infants show no preference for the trochaic or iambic stress pattern by 6 months (in the absence of variability, Höhle et al., 2009). However, bilingual 6-month-olds learning French and German demonstrate a trochaic preference comparable to that of German-learning monolinguals (in the absence of variability, Bijeljac-Babic et al., 2016). Similarly to French-learning monolinguals, Spanish-learning and Catalan-learning infants show no preference for either the trochaic or iambic pattern, both at 6 and 9 months of age (in the presence of variability, Pons and Bosch, 2007). In all the previous studies, preference for stress patterns was tested with CV.CV word shapes. Unlike French-, Spanish-, and Catalan-learning infants, English-learning infants develop a trochaic preference between 6 and 9 months of age (in the presence of variability, Jusczyk et al., 1993). English-learning infants are thus similar to German-learning infants in their stress preferences. A different preference is shown by Hebrew-learning 9-month-old infants, who prefer iambic over trochaic words (in the presence of variability, Segal and Kishon-Rabin, 2012). In summary, three patterns have been found: a listening preference for trochaic stress (English and German), a listening preference for iambic stress (Hebrew), and no preference (French, Catalan, and Spanish). It thus appears that emerging preferences for stress patterns are language-specific and reflect, like discrimination abilities, the acquisition of prosodic properties of the native language. This raises the question of what language-specific properties are driving emerging asymmetries in stress perception or stress preferences.

The divide between variable stress languages, on the one hand, and languages with fixed stress or no lexical stress, on the other, is not enough to explain the preference patterns. Infants learning variable stress languages, like Catalan and Spanish, show no preferred pattern, like learners of a language with no lexical stress, French. Rhythm has been proposed to guide early language acquisition, constraining language discrimination, and early word segmentation abilities (e.g., Nazzi et al., 1998, 2006). In particular, learners of a stress-timed language start by segmenting a trochaic stress unit, given that this is the basic rhythmic unit of the language. Learners of a syllable-timed language use the syllable as the early segmentation unit, as the syllable is the basic rhythmic unit of the language (Nazzi et al., 2006; Butler and Frota, 2018, for a review). This suggests that the rhythmic properties of the language may constrain the early identification and learning of word-forms, and thus the emergence of stress preferences (Höhle et al., 2009). Indeed, learners of stress-timed languages (English and German) develop a preference for the trochaic pattern, which matches the rhythmic trochaic unit, whereas learners of syllable-timed languages (French, Catalan, and Spanish) show no preference, given that the rhythmic unit is the syllable. This view places rhythm at the core of the development of stress perception. According to the rhythmic account, developing stress perception is guided by the acquisition of the rhythmic unit. Learners of stress-timed languages are thus expected to develop a trochaic asymmetry or preference, and learners of syllable-timed languages to develop no asymmetrical perception or preference.

Another possible explanation for emerging stress preferences relates to the frequency of stress patterns in the language. It is expected that infants develop a preference for the dominant pattern of the language. For example, in English, most disyllabic words are trochaic (around 90%, Cutler and Carter, 1987). In Spanish and Catalan, although the trochaic pattern predominates (around 60%), the difference between trochaic and iambic disyllabic words is smaller than in English (Pons and Bosch, 2007, 2010). In Hebrew, iambic stress is the predominant pattern among disyllabic words (around 75%, Segal and Kishon-Rabin, 2012). This suggests that the frequencies of trochaic and iambic patterns are not distant enough in Spanish and Catalan, unlike in English and Hebrew, to trigger the emergence of an early preference. However, in Spanish, most CVC.CV words are trochees (95%), whereas most CV.CVC words are iambs (93%). Interestingly, stress preferences were found to be modulated by word shape. Spanish-learning infants when tested with CVC.CV and CV.CVC word-forms revealed, respectively, a trochaic and iambic preference, while no preference emerged with CV.CV items (Pons and Bosch, 2010).

Finally, the acoustic cues for stress could also explain stress preferences. Pitch, intensity, and duration are the main prosodic cues for stress. It has been shown that trochaic groupings are signaled by increased pitch and intensity on the initial element, while iambic groupings are signaled by increased duration on the final element (Bion et al., 2011; Peña et al., 2016). Thus, trochaic stress tends to be manifested by different acoustic cues (or different cue weighing) from iambic stress, and infants may especially attend to the type of cue(s) that signals stress in the native language (Yoshida et al., 2010; Segal and Kishon-Rabin, 2012). For instance, Hebrew-learning infants preference for iambic stress in the native language did not transfer to the listening of English words, where a trochaic preference was found instead, suggesting that preference is indeed linked to the type of acoustic cues (Segal and Kishon-Rabin, 2012).

The main finding of previous research is that word stress perception is language-specific, and seems to develop as a function of the phonological grammar (presence or absence of lexical stress and the variability in the position of stress) and the prosodic features of the native language (namely, rhythm), together with the frequency distributions and the phonetics of stress. Previous studies have tested infants’ stress perception in languages which are either clearly stress-based (like English and German) or syllable-based (like French, Spanish, and Catalan), or in languages where the frequency distributions of stress patterns provide clear data for a predominant pattern (like English and Hebrew). Studying stress perception in infants learning a language with a mixed prosodic profile, and where the frequency distributions of the trochaic and iambic patterns may not offer sufficient data to establish a clearly dominant pattern, will advance current knowledge of how early stress perception develops. EP is such a language, and thus in the current study, we investigate stress perception in EP-learning infants.

### Stress in European Portuguese

EP has variable stress, similarly to English, Spanish, Catalan, and Hebrew (but unlike French or Finnish). Stress may fall within the last three syllables of the prosodic word and it can distinguish between lexical items (e.g., *bambo* [ˈbɐ̃bu] / *bambu* [bɐ̃ˈbu], “lax” / “bamboo”; *explícito* [ʃˈplisitu] / *explicito* [ʃpliˈsitu], “explicit” / “I make explicit”). It is thus expected that EP-learning infants, who are learning a variable stress language, might develop stress discrimination abilities in the presence of segmental variability, as well as some kind of asymmetrical perception or stress preference (Table 1, first row). However, EP has been shown to have a mixed prosodic profile that combines both stress-timed and syllable-timed rhythm (Frota and Vigário, 2001), differently to other previously studied languages that present either stress-timed (English and German) or syllable-timed properties (French, Spanish, and Catalan). The mixed rhythmic properties of EP may affect the development of stress perception. On the one hand, the presence of stress-timed rhythm may lead to a trochaic preference and allow stress perception to develop similarly to learners of stress-timed languages. On the other hand, the presence of syllable-timed properties may suggest that EP-learning infants develop similarly to learners of syllable-timed languages and show no stress preferences (Table 1, second row). Rhythm perception studies with adults suggest that syllable-timed properties are the most salient ones, as adults are able to discriminate EP from Dutch, which is a stress-based language, on the basis of prosodic cues only (Frota et al., 2002). This finding may be taken as an indication that EP could be perceived as a syllable-based language also by infants. However, emerging word segmentation abilities in EP-learning infants develop differently from learners of syllable-timed languages, suggesting that EP-learning infants are not able to use the syllable as the major rhythmic unit, unlike Spanish-, Catalan-, and French-learning infants (Butler and Frota, 2018).

**Table 1:** Features of European Portuguese (EP) and predictions for the development of stress perception in the first year of life.

Language features	Predictions
Variable/unpredictable stress	Stress discrimination/stress preference
Mixed rhythmic profile Stress-based Syllable-based	Trochaic preference No preference
Frequency of stress patterns Lexical patterns only Plus clitics and monosyllabic words	Trochaic stress Iambic stress
Correlates of stress With vowel quality cues Without vowel quality cues Duration	Stress discrimination Stress “deafness” Iambic stress

The frequency distribution of stress patterns in EP does not consistently pinpoint a given pattern as the dominant one. The frequency of trochaic disyllabic words in EP varies between 66 and 74% in adult speech and 63 and 70% in child-directed speech (depending on whether tokens or types are being considered; data from the FrePoP database, Frota et al., 2010). EP is thus placed roughly between English and Spanish. The higher percentage of trochaic stress compared with Spanish (although not as high as English) suggests that an asymmetrical processing of stress favoring the trochaic pattern would be evident for EP-learning infants. Indeed, the amount of trochaic stress in EP is close to the amount of iambic stress in Hebrew, and Hebrew-learning infants demonstrated an iambic preference (Segal and Kishon-Rabin, 2012). Moreover, the traditional dominant view in the literature on EP assumes that penultimate stress is the common stress pattern (Viana et al., 1996). It is the case, however, that EP has a fair number of unstressed words that depend on other words, also called clitics (around 30% of all word tokens are clitics, Viana et al., 1996; Frota et al., 2006), and of monosyllabic stressed words (around 29%, token frequency, Frota et al., 2006; Vigário et al., 2006a). The great majority of clitics depend on the following word (Vigário, 2003), that is they are unstressed syllables that adjoin to the following stressed word (as in *Vemos o cão* “(We) See the dog”, where the clitic *o* adjoins to the monosyllabic stressed word *cão*, yielding the postlexical word *o cão* that displays iambic stress). In addition, monosyllabic words share many properties with stress-final syllables (Vigário et al., 2006b). Thus, both proclitics and monosyllables may add to the iambic stress patterns of the language, thus reducing the distance between trochaic and iambic stress. If these elements are taken into account, iambic stress becomes more frequent than trochaic stress (Vigário et al., 2010). It is thus possible that the frequency distributions of stress patterns are not clear enough to highlight one pattern over the other (Table 1, third row).

The correlates of stress in EP also offer a diverse set of cues that combine prosodic and segmental cues. Pitch has not been regarded as a correlate of word stress in the literature on EP stress. This is not surprising given the low co-variation between stress and intonational pitch movements (or pitch accents). In EP, most stressed syllables do not get a pitch accent, and intonational pitch movements are found mostly in the most prominent syllable of the utterance, typically in the final word (Frota, 2002, 2014). EP is thus unlike English, and other trochaic grouping languages, with respect to the low weight of pitch as a cue for stress (Chrabaszcz et al., 2014; Zahner et al., 2016). By contrast, duration has been reported to be the main cue for word stress in EP, particularly in the absence of vowel quality cues (Delgado-Martins, 1977, 1986; Andrade and Viana, 1989). The role played by duration as a strong prosodic correlate of stress in EP, whereas pitch has a negligible role, is thus similar to Hebrew, a language where iambic stress has been reported to predominate (Segal and Kishon-Rabin, 2012; Silber-Varod et al., 2016). However, recent research has shown that vowel quality cues are the primary cues for stress perception in EP. Phonological vowel reduction is a general phenomenon in the language affecting all unstressed positions (with few exceptions), so that the contrast between low, mid, and high vowels found in stressed syllables (/i, e, ɛ, a, u, o, ɔ/) does not hold in unstressed syllables. In these syllables, over 90% of all vowels are those that belong to the reduced vowel system, i.e., [i, ɨ, ɐ, u] (data based on the FrePoP Lexicon, Vigário et al., 2015). Behavioral findings from adult perception have shown that, in the absence of vowel quality cues, EP speakers are unable to perceive stress contrasts, demonstrating a stress “deafness” effect similar to that found in speakers of languages with fixed stress or no lexical stress (Correia et al., 2015; Lu et al., 2018). In the presence of vowel quality cues, stress contrasts are clearly perceived. These findings suggest that duration is not a sufficient cue for stress in EP and that vowel reduction has taken over the prosodic cues. Interestingly, in an ERP study, EP adult speakers were able to discriminate stress patterns in the absence of vowel quality cues, showing that the requirements of stress processing may be different at the pre-attentive (mismatch negativity, MMN) and attentive stages (Lu et al., 2018). In addition, both the behavioral and ERP results have shown an asymmetrical stress perception with a processing advantage for iambic stress (more accurate and more quick responses, more negative ERP components; Lu et al., 2018). Taken together, previous findings on the correlates of stress and stress perception highlight particular features of EP. The correlates of stress are an uncommon combination of longer duration in stressed syllables, vowel reduction in unstressed syllables, and low co-variation between stress and pitch, which does not match English, Spanish, Catalan, or Hebrew. Also unusual is the finding of stress “deafness” in a variable stress language, together with a processing advantage for iambic stress, in the absence of vowel quality cues and in the presence of prosodic cues, namely duration (Table 1, fourth row).

To date, there have been no previous studies into EP-learning infants’ developing stress perception abilities. It is thus unknown whether the adult perception findings extend to infants. In a word learning study that included a stress contrast condition (e.g., /ˈmilu/ vs. /miˈlu/), 1–4-year-old children demonstrated sensitivity to stress location not recognizing a word as the learned word if it only differed in the stress pattern (Frota et al., 2012). While this provided some indication that infants and toddlers are able to distinguish between stress patterns, a stress perception study needs to be conducted to ascertain early stress perception abilities in EP-learners. In the present study, we are looking at stress perception at 5–6 months of age. This age was chosen for several reasons. First, discrimination, asymmetrical perception, or preference in the presence of segmental variability have not been evident before 8 months in previous studies. Most previous studies, however, have used behavioral methodologies, i.e., variants of the Headturn Preference Procedure (HPP), which may not be a sensitive enough method to capture effects in this domain before 8 months. Our study utilizes an eye tracking methodology that is potentially more sensitive to investigate infants’ perceptual and cognitive abilities (Gredebäck et al., 2010; Francois et al., 2018). Secondly, preference or asymmetry in stress perception in the absence of segmental variability emerges after 4 months of age in some languages (Weber et al., 2004; Friederici et al., 2007; Höhle et al., 2009). Thirdly, language-specific perception in the pitch domain has been shown as early as at 4–5 months (Yeung et al., 2013; Frota et al., 2014; Sundara et al., 2015). Finally, stress-related early markers of risk for later language impairments have been demonstrated to be evident at 5 months of age (Weber et al., 2005; Friedrich et al., 2009). Studying stress perception in EP-learning infants will provide new data contributing to the understanding of the role of native phonological grammar, rhythm, frequency, and stress cues in how stress perception develops in language acquisition. The predictions according to the particular features of EP have been summarized in Table 1. Most importantly, the ability to distinguish stress patterns in the absence of vowel quality cues (through discrimination, asymmetrical perception, or preference) would indicate that early perception is guided by prosodic cues, not segmental cues, unlike adult perception. Rhythmic properties as well as frequency of stress patterns, lead to different predictions about (a)symmetry in stress perception, depending on the specific rhythmic feature that may be most salient and the way lexical and non-lexical word patterns are included in or excluded from frequency computations. Finally, the prosodic correlates of stress suggest an asymmetry favoring iambic stress. However, it is unclear which of these factors most influences early stress perception.

## Materials and Methods

### Participants

Twenty-four infants participated in this study (16 males, mean age 5 months 26 days, range 5 months 1 day–6 months 27 days). All were typically developing infants raised in monolingual EP homes, recruited from the wider Lisbon area. Seven additional infants were tested but excluded due to fussiness (2), poor tracking (i.e., overall tracking ratio below 30%, 4), and an autistic close family member (1). All caregivers completed the CSBS-DP Checklist (a developmental screening tool – Wetherby and Prizant, 2002) adapted for EP, and included infants demonstrated overall social communication, language, and symbolic functioning skills as expected for their age range (including eye gaze, gestures, use of sounds, and understanding) when compared with the means and standard deviations of the English standardization sample (Table 2).

**Table 2:** CSBS-DP Checklist scores for the infants included in the study compared with the standardized scores for similar age group infants.

*n*	Social composite		Speech composite		Symbolic composite		Total	
	*M*	*SD*	*M*	*SD*	*M*	*SD*	*M*	*SD*
24	10.83	2.82	3.29	1.33	3.71	1.9	17.83	4.79
50	10.00	2.95	3.74	1.76	4.32	1.48	18.06	4.7
Cut-off	>8	–	>2	–	>3	–	>13	–

We defined our sample size of 24 infants on the basis of previous studies (e.g., Skoruppa et al., 2009; Abboub et al., 2015; Bruderer et al., 2015) with similar designs, and their reported large effect sizes (Lakens, 2013). Considering an estimated effect size of 0.16 (defined on the basis of previous studies), and a design with three repeated measures with two levels each, a sample size of 24 would result in a power of 0.82 (using the fpower tool in Stata 15, StataCorp. 2017, Stata Statistical Software: Release 15, College Station, TX: StataCorp LLC; Ender, 1995).

This study was carried out in accordance with the recommendations of the European Union Agency for Fundamental Rights and the Declaration of Helsinki, with informed written consent obtained from caregivers prior to data collection. The study was approved by the ethics committees “Comissão de Ética para a Saúde do Centro Hospitalar Lisboa Norte” (Ref.ª DIRCLN-16JUL2014-208) and “Comissão de Ética para a Saúde da Administração Regional de Saúde de Lisboa e Vale do Tejo” (Proc.015/CES/INV/2014).

### Materials

**Figure 1:**
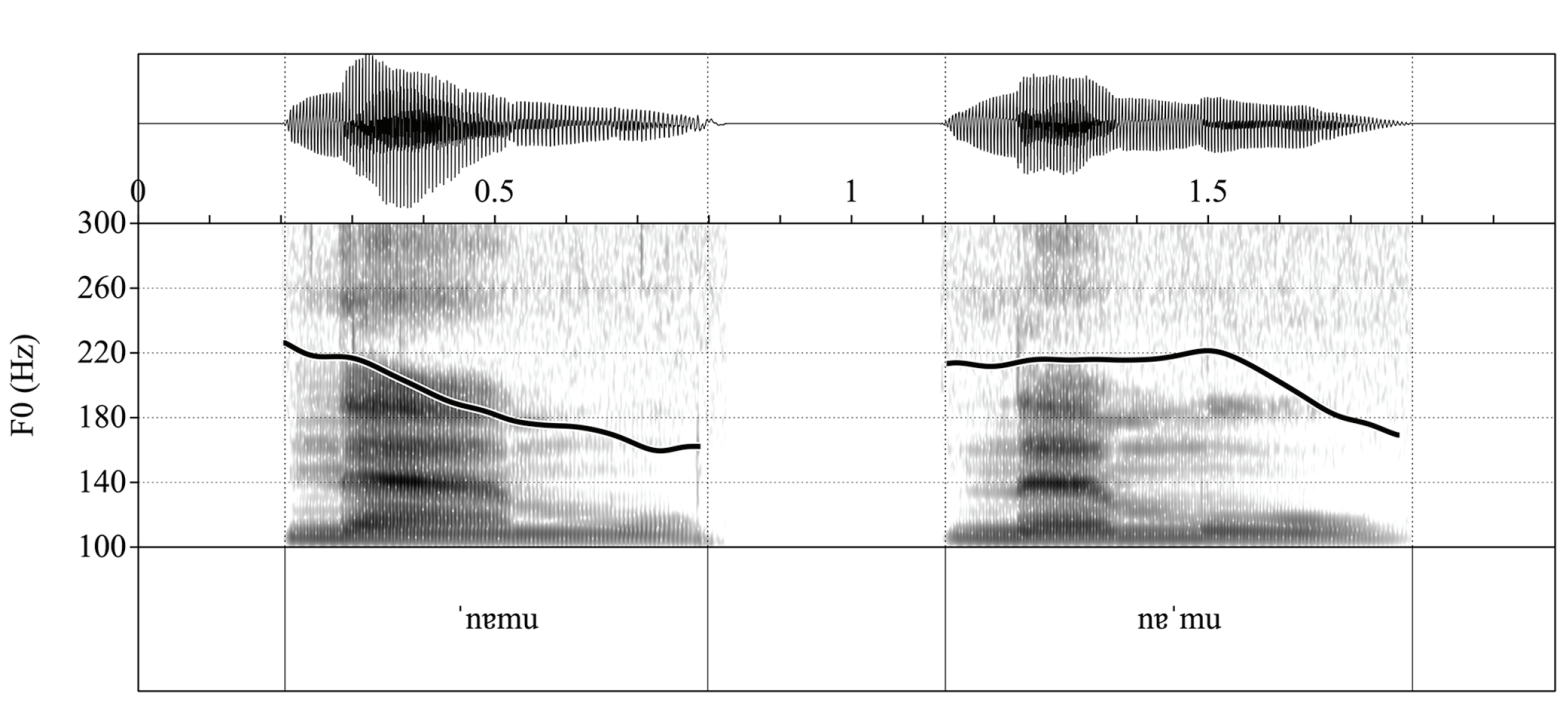
Pitch contours for trochaic stress (pitch fall on the penult syllable) and iambic stress (pitch fall on the final syllable).

**Figure 2:**
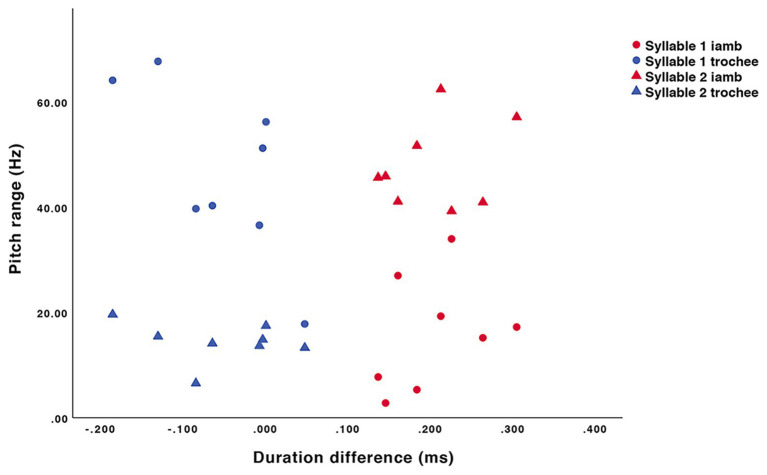
Pitch range (in Hertz), and differences in duration (in milliseconds) between the final syllable (syllable 2) and the initial syllable (syllable 1) in trochaic and iambic stimuli.

A set of eight disyllabic, segmentally varied pseudo-words was used with stress in initial (trochaic) and final (iambic) positions, uttered by a female speaker in child-directed speech. The structure of the pseudo-words was as follows: C_1_V_1_C_2_V_2_ (i.e., [‘milu] / [mi’lu], [‘tɐnu] / [tɐ’nu]). Consonants were selected from the most-used consonants in Portuguese. Stops, fricatives, and liquids were balanced. Both in training and testing, there were four stops, one nasal, one fricative, and one liquid. Within a training or test sequence, C_1_ and C_2_ were different between words. V_1_ ([ɐ], [i], or [u]) was balanced across training and testing. These vowels were used because they are the only ones that may appear both in stressed and unstressed positions. V_2_ was always [u], given that this is the only vowel that may frequently appear both in word-final unstressed and stressed positions. Thus, no vowel quality cues to stress were present in the stimuli. Prosodic cues were the only cues to stress: the duration of the stressed syllable was longer, and the location of the pitch fall signaled the stressed syllable (given that the pseudo-words were produced as single-word utterances). Figure 1 shows the pitch contours for iambic and trochaic stress patterns, while Figure 2 demonstrates the pitch range and duration differences evident in the stimuli. These differences between the iambic and trochaic pseudo-words were all significant [duration, initial syllable: *t*(14) = −5.459, *p* < 0.001, *d* = 2.73, final syllable *t*(14) = 6.383, *p* < 0.001, *d* = 2.21; pitch range, initial syllable *t*(14) = −4.416, *p* < 0.01, *d* = 3.19, final syllable *t*(14) = 10.353, *p* < 0.001, *d* = 5.18].

### Procedure

An adaptation of the anticipatory eye movement (AEM) paradigm (McMurray and Aslin, 2004) was used to examine infants’ perception of stress utilizing eye-tracking. We trained infants using two cuing visual stimuli to make two different responses that consisted in looking to the left or right side of the screen, and we used their ability to anticipate the appearance of visual stimuli. Similarly to Richardson and Kirkham (2004), we presented infants with blocks of training and test, instead of a long training and then a test phase, to maximize the data collected from participants given their young age. The experiment was conducted in a dimly lit and sound attenuated laboratory. Infants were sat in an appropriate, child-friendly high chair, or on their parent’s lap, in front of a Dell LCD screen (1,680 × 1,050 pixel resolution) of an RED SensoMotoric Instruments (SMI) eye-tracker, on which they viewed the images, while speakers concealed behind the screen played the recorded speech stimuli. The child was situated approximately 70 cm from the screen. The presentation of the stimuli and the storing of the child’s eye movement data were performed with the SMI Experimenter Center and iView X software. A camcorder mounted above the screen allowed the experimenter to monitor the participants’ behavior (*via* the Observation Software package from SMI).

The session began with a two-point infant calibration, followed by the experimental procedure. Each trial begin with a fixation point in the middle of the screen, and trials began once the infant fixated this point for 400 ms. The experiment consisted of two phases: training and testing. During training trials, infants were presented with two frames on either side of the screen, with an animated image (either a moving triangle or cross) inside one of the frames, while one of the stress patterns (either iambic or trochaic) was presented through the speakers. In a training trial, four different pseudo-words with the same stress pattern were presented with a 1,200 ms inter-stimulus interval. In total, there were six training trials, three trials with iambic sound stimuli and three trials with trochaic sound stimuli. The side of presentation of the image was linked to one of the sounds, for example, all iambic trials on the left and all trochaic trials on the right. One of the images was paired with one sound type, e.g., triangle with iambic, cross with trochaic. The total duration of each training trial was 7,000 ms. Auditory stimuli onset aligned with the beginning of the trial and the offset was around 6,000 ms from trial beginning. During the test phase, infants were presented with two test trials. During each trial, the image contained the two frames in the same position as in the training but without the animated image. In one of the test trials, the iambic sound stimuli were played and in the other test trial, the trochaic sound stimuli were presented. The sound stimuli for each test trial included four different pseudo-words with the same stress pattern, presented with a 1,200 ms inter-stimulus interval. The total duration of each test trial was 7,000 ms, and auditory stimuli onset-offset time was the same as for training trials. If infants had learnt during the training phase to associate the stress pattern heard to the side of the screen the animated image appeared, during the test phase they should look to the side of the screen where the sound stimuli presented cues the infant to look.

The side of presentation and image associated with the sound type was counterbalanced across participants. Presentation of training trials was pseudo-randomized, so that infants were not presented with more than two training trials of the same type in a row. The sound stimuli in each trial contained four pseudo-words, and the pseudo-words were different for training and test.

**Figure 3:**
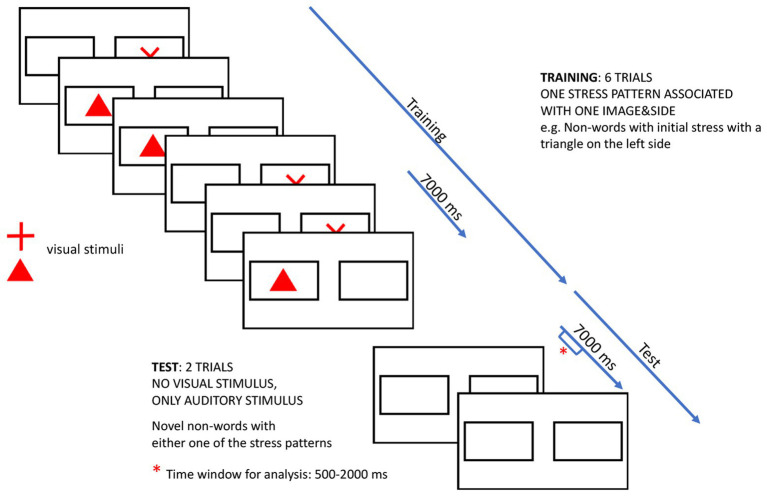
Structure of an experimental block.

The six training and two test trials made up one block of the experiment, and the structure of a block can be seen in Figure 3. The experiment consisted of eight blocks, and it continued until all eight blocks were presented or the infant lost interest in the experiment. Infants only needed to complete one block to be included. The colors of the animated images were the same within a block (e.g., red triangle and red cross), and were changed between blocks to refresh the infants’ interest in the images. The last training trial/first test trial was also controlled for any effect of hearing the same stress pattern between the two. This alternated between blocks (same/different), and the order was counterbalanced across infants (same first block/different first block). Between each block, a reward video was presented, which consisted of an animated cartoon character (Noddy), saying one of four phrases: “É isso! Vamos jogar mais uma vez” (That’s it! We are going to play one more time), “Muito bem! Vamos continuar o nosso jogo” (Well done! We are going to continue our game), “Muito bem! Este jogo é muito divertido” (Well done! This game is a lot of fun), and “Parabéns! Vejo que estás mesmo a gostar disto” (Good! You are really enjoying the game). Presentation of the reward videos was randomized across infants. Sound files for training and test trials, together with a sample video showing the last training trial, the two test trials and a reward video from one experimental block, are available at http://labfon.letras.ulisboa.pt/babylab/Infants_Perception/Infants_perception_stress_supporting_materials.htm.

We expect infants to look at the iambic trained side while listening to an iambic test trial and at the trochaic trained side while listening to a trochaic test trial in case they are sensitive to contrasting stress patterns. There is another possibility, which is that the infants will show an asymmetry and look more at one of the trained sides regardless of the stimuli heard during the test trials. This would show that besides differentiating between the stimuli they have a preference for one of the stress patterns.

## Results

Infants completed between two and six blocks (mean 4), and thus completed between four and 12 test trials (mean 8). For three of the infants, the two test trials from a block had to be excluded due to technical error. However, these infants were included in the analysis as they completed between two and five blocks (i.e., between four and 10 test trials). For the analysis, areas of interest were defined that related to half of the screen, rather than just analyzing the frame areas. As the infants were quite young, this controls the possibility that, when there is no image to focus on during test trials, the infants may look to the side of the screen rather than within a particular frame. Looking time was defined as the net dwell time, i.e., the sum of sample durations of all gaze data samples within the area of interest during a given time window (SMI Begaze manual, version 3.7, December 2016, p. 368).

**Figure 4:**
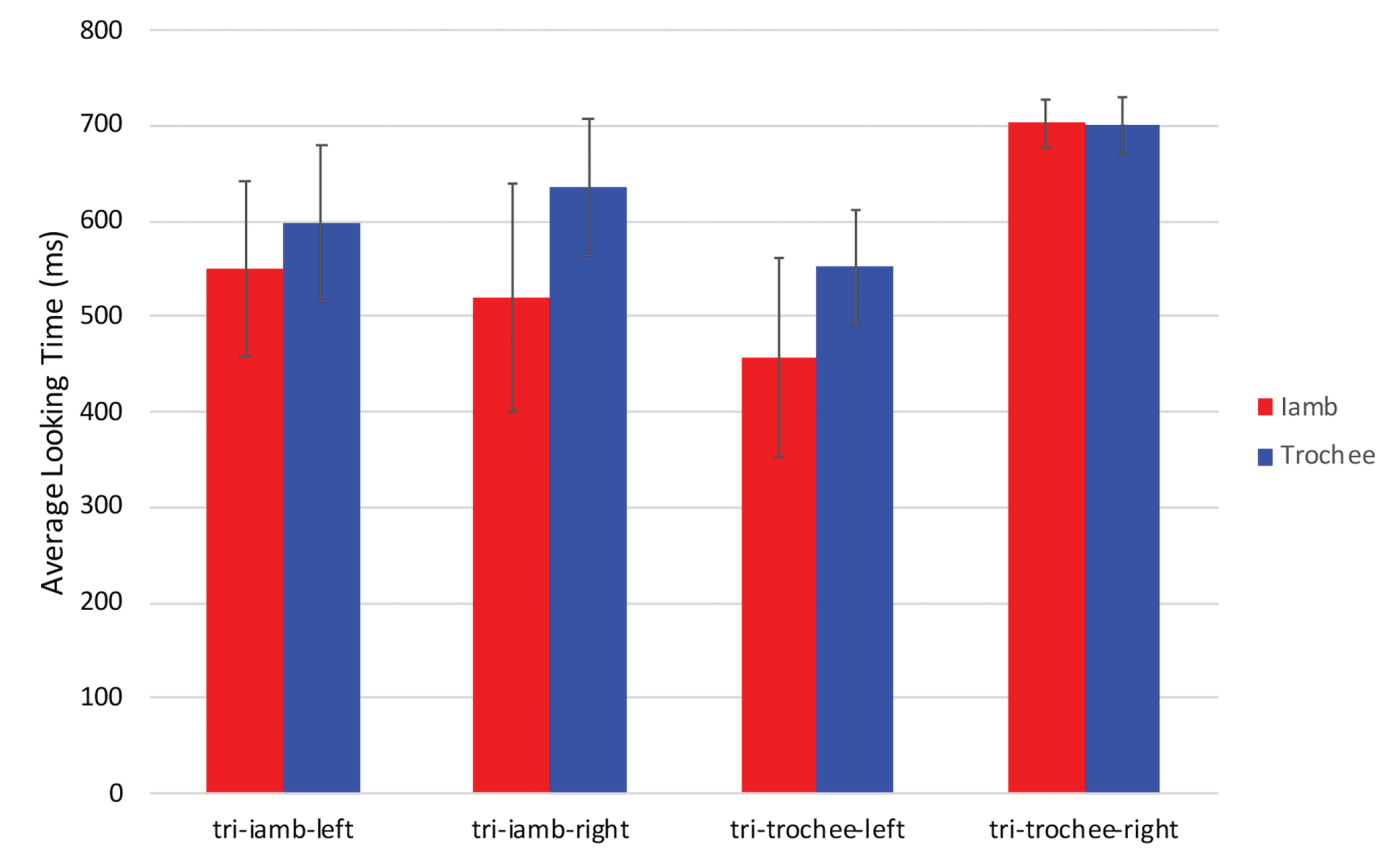
Average looking times (in milliseconds) during the training phase, considering side and image. Error bars indicate the standard error of the mean (±1).

First the training phase was analyzed to compare looking times during the iambic and trochaic trials and to test for an effect of side and image association (four conditions – tri-iamb-left, tri-iamb-right, tri-trochee-left, and tri-trochee-right). Figure 4 shows the average looking times to the animated image during training trials across the four conditions. A 2 (training trial) × 4 (condition) ANOVA revealed no difference in looking times between iambic and trochaic training trials [*F*(1,20) = 2.8, *p* = 0.11, η^2^*_p_* = 0.12], no effect of condition [*F*(3,20) = 1.11, *p* = 0.37, η^2^*_p_* = 0.14], and no interaction between the two factors [*F*(3,20) < 1].

**Figure 5:**
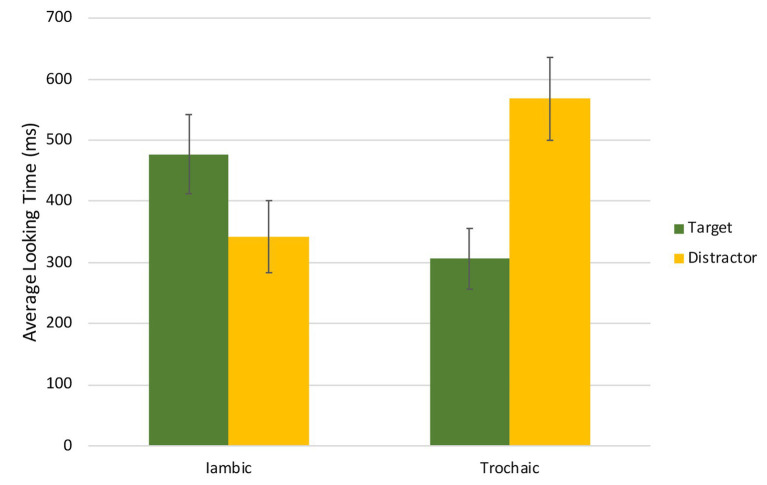
Average looking times (in milliseconds) to the target and distracter sides for iambic and trochaic test trials. Error bars indicate the standard error of the mean (±1).

We then examined infants’ looking behavior during the test phase. We selected a time window 500 ms after trial onset until 2,000 ms, allowing the infants sufficient time to process the sound stimuli and initiate an eye movement, as well as account for the infant losing interest once the animated image did not appear. For each test trial, we calculated the looking time during this window to the target side (i.e., the trained side cued by the sound stimuli in test trials) and the distracter side, and we took into account the order of the last training trial/first test trial (same/different) and the stimuli that was heard during test trials (iambic/trochaic). Figure 5 shows the looking times to the target and distracter sides for iambic and trochaic test trials. The data were first analyzed using a 2 (Side) × 2 (Order) × 2 (Stimuli) ANOVA. We found no effect of Side [*F*(1,23) = 2.42, *p* = 0.13, η^2^*_p_* = 0.09], Order [*F*(1,23) = 2.18, *p* = 0.15, η^2^*_p_* = 0.09], or Stimuli [*F*(1,23) < 1], but there was a significant interaction between Side and Stimuli [*F*(1,23) = 6.06, *p* = 0.02, η^2^*_p_* = 0.21]. The interaction between Side, Stimuli, and Order was borderline significant [*F*(1,23) = 4.97, *p* = 0.04, η^2^*_p_* = 0.18], reflecting the fact that differences in looking time across side and stimuli were higher when the last training trial and the first test trial were different. All other interactions were not significant.

**Figure 6:**
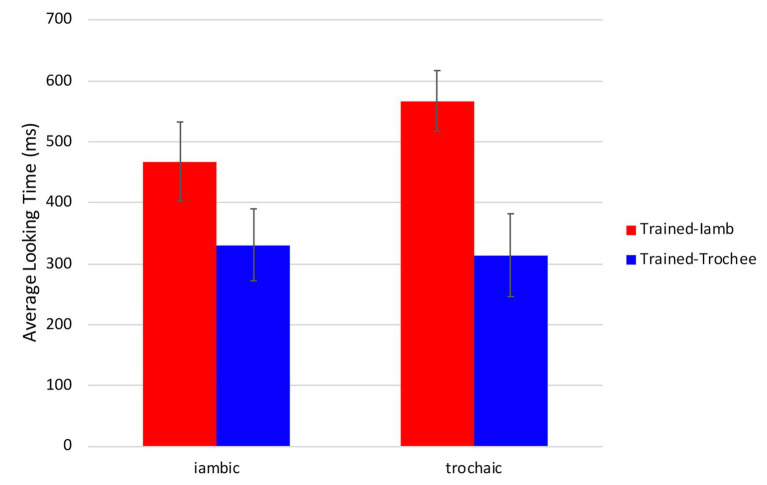
Average looking times (in milliseconds) to the iambic and trochaic trained side during iambic and trochaic test trials. Error bars indicate the standard error of the mean (±1).

Importantly, the interaction between Side and Stimuli suggests an asymmetry in infants’ looking behavior. To investigate this further, we re-coded the looking times in the test trials so that, instead of target and distracter side, we analyzed the iambic trained side and the trochaic trained side, regardless of the stimuli heard during the trial. For example, if iambic stress was trained to the left side, we coded looking to the left of the screen during all test trials as iambic. Figure 6 shows the looking times to the iambic and trochaic trained side during iambic and trochaic test trials. Overall, infants looked longer at the iambic trained side (517 ms) than at the trochaic side (323 ms). A 2 (Trained side) × 2 (Order) × 2 (Stimuli) ANOVA revealed a significant effect of trained side [*F*(1,22) = 6.14, *p* = 0.02, η^2^*_p_* = 0.22], with no effect of Order [*F*(1,22) = 3.10, *p* = 0.09, η^2^*_p_* = 0.12] or Stimuli [*F*(1,22) = 1.74, *p* = 0.20, η^2^*_p_* = 0.07], and no significant interactions.

Given that participants contributed to our findings with different numbers of test trials, infant looking times in the test phase were further analyzed using generalized linear mixed models in SPSS version 26.0 (IBM SPSS Statistics). Two analyses were run. In both, we controlled for participants in the random effect structure, we used a Satterthwaite approximation to account for differences across groups, and a robust estimation of the covariances to account for small sample sizes. The first analysis examined looking time to target and distracter. The fixed effects included Side (Target, Distracter), Order (Same, Different), Stimuli heard during the test trial (iambic, trochaic), and their interaction. There was no effect of Side [*F*(1,336) = 2.27, *p* = 0.13], Order [*F*(1,348) = 2.28, *p* = 0.13], or Stimuli [*F*(1,336) < 1]. There was a strong interaction between Side and Stimuli [*F*(1,336) = 17.15, *p* < 0.001; *ß* = 581.37, *SE* = 133.77, *t* = 4.35, *p* < 0.001]. The interaction between Side, Stimuli, and Order was also significant [*F*(1,336) = 4.588, *p* = 0.03; *ß* = −395.71, *SE* = 185.20, *t* = −2.14, *p* = 0.03]. No other significant effects were found. The second analysis investigated the asymmetry in infants’ looking behavior suggested by the first analysis. It thus examined looking time to the iambic trained side and the trochaic trained side, regardless of the stimuli heard during the test trial. The fixed effects included Trained Side (Trained-iamb, Trained-trochee), Order (Same, Different), Stimuli heard during the test trial (iambic, trochaic), and their interaction. The model revealed a significant effect of Trained side [infants looked longer at the iambic trained side, *F*(1,336) = 16.98, *p* < 0.001; *ß* = 380.76, *SE* = 93.98, *t* = 4.05, *p* < 0.001], with no effect of Order [*F*(1,348) = 1.88, *p* = 0.17] or Stimuli [*F*(1,336) = 1.39, *p* = 0.24], and no significant interactions. The linear mixed model analysis also provided evidence that infants’ looking behavior is asymmetrical with a preference for the iambic stress pattern.

## Discussion

In this study, we investigated infants’ stress perception abilities in a previously unstudied language, EP. Unlike the languages where infant stress perception has been formerly tested, EP is neither clearly stress-based (as English and German) nor syllable-based (as French, Spanish, and Catalan). In addition, the language does not offer a straightforward difference in the frequency of trochaic and iambic stress patterns (unlike English or Hebrew). Using a version of the AEM paradigm (McMurray and Aslin, 2004) implemented with eye-tracking, infants at 5–6 months were shown to be sensitive to the trochaic/iambic stress contrast, with evidence of asymmetrical perception or preference for one of the stress patterns, namely infants’ looked longer at iambic stress. Our findings provide the first evidence of asymmetrical perception or stress preference by 6 months of age in the presence of segmental variability, a more challenging context for young learners that requires the ability to abstract and generalize stress patterns across the phonetic variability shown by the stimuli.

As learners of a variable stress language (where stress is used contrastively), EP-learning infants were expected to develop their stress processing skills and show sensitivity to the different stress patterns in the presence of segmental variability at some point in the first year of life, like English-, Spanish-, Catalan-, and Hebrew-learning infants (Jusczyk et al., 1993; Skoruppa et al., 2009, 2011; Segal and Kishon-Rabin, 2012). This sensitivity has already emerged by 5–6 months in the case of EP-learning infants. While this result is in line with previous infant studies on variable/unpredictable stress languages, it reveals important differences between infant and adult stress perception in EP. In the current study, infants’ perception was tested in the absence of vowel quality cues to stress. Findings from behavioral studies showed that without the vowel quality cues EP adult speakers do not perceive stress contrasts, demonstrating a stress “deafness” effect similar to that found in speakers of languages with fixed stress or no lexical stress (Correia et al., 2015; Lu et al., 2018). This suggests that vowel reduction has taken over the prosodic markings of stress in adult speakers’ phonological grammar (Rahmani et al., 2015; Lu et al., 2018). The ability to distinguish stress patterns in the absence of vowel quality cues demonstrated by 5–6-month-olds indicates that early perception is guided by prosodic cues, not segmental cues, unlike adult perception. Thus, prosodic cues are sufficient for infant stress perception, and more language experience seems to be required to develop stress “deafness” in the absence of vowel reduction. The finding that 1–4-year-old children demonstrated sensitivity to stress location in a word learning study (Frota et al., 2012) suggests that by 4 years of age children’s phonological grammar regarding stress is not yet like adult grammar. It is possible that the developmental change from prosodic to vowel quality cues in stress perception is related to the acquisition of vowel reduction. Child production data have shown that unstressed vowels are beginning to be acquired around 2 years of age and that, at least for some unstressed vowels, acquisition is still ongoing around 3 years of age (Freitas, 2007). Mastering reduced vowels is required to establish the contrast between a full (stressed) vowel and a reduced (unstressed) vowel (as in *chapa* [ˈʃapɐ], “plate” vs. *chapéu* [ʃɐˈpɛw], “hat”; [paˈpɛw], a common child production of *hat* reported in Freitas (2007), shows a full vowel in the unstressed syllable). It is precisely this contrast that offers the strong vowel quality cues for stress in the language. It is thus expected that vowel quality cues may take over prosodic cues only after vowel reduction is acquired. The point in development when children no longer attend to prosodic cues in stress perception needs to be investigated in future research. Although infant and adult stress perception differ with regard to sensitivity to prosodic cues, it is important to highlight that they share a common feature: a processing advantage for iambic stress. Both the behavioral and ERP results on adult stress perception have shown an asymmetry favoring iambic stress (Lu et al., 2018). Similarly, the present findings on infant perception demonstrated evidence of asymmetrical perception favoring iambic stress.

The rhythmic properties of the native language have been proposed to affect the development of stress perception (Nazzi et al., 2006; Höhle et al., 2009). Specifically, learners of stress-based languages (English and German) develop a preference for the trochaic pattern, which relates to the acquisition of the trochaic rhythmic unit, and learners of syllable-based languages (French, Catalan, and Spanish) develop no preference, which is explained by the acquisition of the syllable as the rhythmic unit. EP displays mixed rhythmic properties combining stress-based and syllable-based features (Frota and Vigário, 2001). Thus, the rhythmic account would predict either a trochaic asymmetry or preference, in case the stress-based features are the most salient (as suggested by findings on emerging word segmentation abilities in EP-learning infants, Butler and Frota, 2018), or no stress preference, in case the syllable-based features are the most salient (as suggested by adult rhythm perception, Frota et al., 2002). Crucially, the EP-learning infants’ asymmetrical perception favoring iambic stress is not predicted by the rhythmic account.

Another factor that has been proposed to drive the early acquisition of word stress is the language-specific frequency distribution of stress patterns. Under this account, infants would develop an asymmetrical perception or preference that corresponds to the acquisition of the predominant lexical stress pattern of the native language (Jusczyk et al., 1993; Weber et al., 2004; Segal and Kishon-Rabin, 2012). For example, German infants’ asymmetrical perception or preference, favoring trochaic stress would be explained by the predominance of the trochaic pattern in the language. Similarly, the predominance of iambic stress in Hebrew would explain the iambic preference demonstrated by Hebrew-learning infants. To our knowledge, the frequency distribution of stress patterns in previous studies has always been established on the basis of lexical words with more than one syllable. If only these words are considered, trochaic stress is the predominant pattern in EP, thus predicting an asymmetrical processing of stress favoring the trochaic pattern, contrary to our findings. However, if monosyllables and clitics are also included in the frequency computations, iambic stress becomes predominant (Vigário et al., 2010), and the opposite asymmetry or preference is expected. The finding of an asymmetry in EP-learning infants’ looking behavior favoring iambic stress could thus be explained by the acquisition of the predominant stress pattern in the language. This strongly suggests that the computation of language-specific frequency patterns for stress needs to go beyond lexical words with disyllabic (or more than one syllable) shapes, taking into account phonological features of the language that may impact the distribution of stress patterns (e.g., cliticization and phonology of monosyllabic words).

Current accounts of developing stress perception have relied mostly on the rhythmic properties of the native language and the predominance of a given stress pattern established on the basis of lexical frequencies (Bhatara et al., 2018). It has been proposed that infants’ perception develops from a general sensitivity to prosodic information into the acquisition of language-specific prosodic properties, and that the crucial factors playing a role in this development are rhythm and the dominant stress pattern. Our findings strongly suggest the need to consider more factors in this process, along with a deeper analysis of the phonological and phonetic features of the language. Proposed candidates are the contributions of lexical and postlexical words, as well as cliticization, to stress patterns, and the types of acoustic dimensions that instantiate stress. These factors may become especially prominent when the rhythmic properties provide conflicting cues (as in the case of EP). However, whether and how the different factors are weighted remains an open question to be addressed in future research targeting more diverse languages.

In conclusion, the present findings demonstrated that EP-learning infants are sensitive to the trochaic/iambic stress contrast, providing the first evidence of sensitivity to stress patterns as early as at 5–6 months of age in the presence of segmental variability. This result reveals the success of the AEM paradigm using eye-tracking in studying infant perception at such young ages. Results from this research have also shown that early stress perception is guided by prosodic cues (duration being the main prosodic cue for word stress), and not segmental cues, unlike adult stress perception. Importantly, EP-learning infants showed an asymmetrical perception favoring iambic stress that was not predicted by the rhythmic account of developing stress perception, but explained instead by phonological patterns that affect the frequency distribution of trochaic and iambic stress, together with the high weight of duration as a stress correlate. This finding calls to more attention to be given to language particular stress-related phonological and phonetic features in studies on the early acquisition of lexical stress. By investigating a type of language not previously tested in the infant stress perception literature, namely a language with a mixed prosodic profile, where conflicting factors impact the frequency distributions of the trochaic and iambic patterns, and the main correlates of stress are a mixture of duration and vowel reduction, the present study sets the stage for future cross-linguistic research on early stress perception in languages with different combinations of rhythmic, phonological, and phonetic properties.

## Data Availability Statement

The raw data supporting the conclusions of this article will be made available by the authors, without undue reservation, on request to the corresponding author.

## Ethics Statement

The studies involving human participants were reviewed and approved by Comissão de Ética para a Saúde do Centro Hospitalar Lisboa Norte and Comissão de Ética para a Saúde da Administração Regional de Saúde de Lisboa e Vale do Tejo. Written informed consent to participate in this study was provided by the participants’ legal guardian/next of kin. Written informed consent was obtained from the minor(s)’ legal guardian/next of kin for the publication of any potentially identifiable images or data included in this article.

## Author Contributions

SF and MV conceived and designed the study. EU implemented the experiment. EU, JB, and CS collected the data. JB, EU, and CS analyzed the data. SF and JB wrote the manuscript. All authors contributed to the article and approved the submitted version.

### Conflict of Interest

The authors declare that the research was conducted in the absence of any commercial or financial relationships that could be construed as a potential conflict of interest.
